# Retrodeformation of fossil specimens based on 3D bilateral semi-landmarks: Implementation in the R package “Morpho”

**DOI:** 10.1371/journal.pone.0194073

**Published:** 2018-03-19

**Authors:** Stefan Schlager, Antonio Profico, Fabio Di Vincenzo, Giorgio Manzi

**Affiliations:** 1 Department Biological Anthropology, University Medical Center, Freiburg, Germany; 2 Department of Environmental Biology, Sapienza Università di Roma, Rome, Italy; Monash University, AUSTRALIA

## Abstract

Many fossil specimens exhibit deformations caused by taphonomic processes. Due to these deformations, even important specimens have to be excluded from morphometric analyses, impoverishing an already poor paleontological record. Techniques to retrodeform and virtually restore damaged (i.e. deformed) specimens are available, but these methods genenerally imply the use of a sparse set of bilateral landmarks, ignoring the fact that the distribution and amount of control points directly affects the result of the retrodeformation. We propose a method developed in the R environment and available in the R-package “Morpho” that, in addition to the landmark configurations, also allows using a set of semi-landmarks homogeneously distributed along curves and on surfaces. We evaluated the outcome of the retrodeformation, regarding the number of semi-landmarks used and its robustness against asymmetric noise, based on simulations using a virtually deformed gorilla cranium. Finally, we applied the method to a well-known Neanderthal cranium that exhibits signs of taphonomically induced asymmetry.

## Introduction and aim

The fossil record of terrestrial vertebrates is notoriously poor and fragmentary and many fossil specimens show, to various extent, some deformations due to taphonomic processes (e.g. shearing, bending, compression) [1–6].

The inclusion of such distorted specimens in morphometric analyses might bias the results randomly or even directionally i.e. simulating idiosyncratic variability (e.g. patterns of asymmetry) or specific evolutionary changes and trends [3,7,8]. However, their exclusion could lead to a significant loss of palaeobiological information given the above-mentioned scarcity of comparative material. Thus, paleontologists are often faced with the “dilemma” whether to exclude an important but deformed specimen from the analyses or to use it adopting some cautions and estimates. This dilemma becomes relevant in particular cases such as studies on human evolution where unique morphologies are often associated with badly preserved fossil specimens [7]. Thus, in order to avoid the undesirable effects of the distortions and to include as much data as possible, paleoanthropologists employ a number of techniques to restore the specimens according to their suspected original morphology. Many of these techniques use the bilateral symmetry of the specimen to perform the integration and replacement of the missing portions (via mirroring) and/or other undistorted specimen(s) as reference(s) to perform the retrodeformation, which is the process of removing distortions in fossils caused by taphonomic forces [9–13].

During the last two decades, the rise of computerized tomography (CT-scan) and 3D modeling techniques, such as laser scanning and photogrammetry, allowed for the application of virtual approaches to restoring the bilateral symmetry of a digital model [14,15]. The standard techniques to virtually restore the missing parts are subsumed under the name “virtual restoration” [16]; the set of protocols to remove the distortion between the right and the left side is called “symmetrization” [17–19]. Such techniques are a common issue in palaeoanthropology [11,20–22] since the virtual reconstruction of the Neanderthal cranium of Le Moustier 1 [20].

A common approach to restore symmetry in a digital model is based on the acquisition of bilateral landmarks. These landmarks are reflected and relabeled in order to compute a symmetric average of both the original and the mirrored and relabeled set of landmarks. Subsequently, the 3D model is warped to the symmetric consensus using a Thin-Plate-Spline deformation (TPS) [23]. Gunz and colleagues employ this approach to remove uniform shearing (Reflect Relabeling, RR) [16,24]. Recently, a non-linear symmetrization (NLS) method to restore bending and/or compression landmark-based was proposed by Ghosh and colleagues [25] and evaluated by Tallman and colleagues [13]. Both methodological approaches are restricted by the number of available landmarks that can reliably be placed on both sides.

We implemented a set of functions into the well-established Morphometrics R-package Morpho [26], to allow an easy-to-use interface for the virtual retrodeformation of fossils, with the possibility of using both curves and semi-landmarks as well as landmarks. The introduction of semi-landmarks in the symmetrization procedures can be useful to acquire some anatomical structures (e.g. the outlines of the foramen magnum, orbits and piriform aperture, the temporal lines) otherwise difficult to define using landmarks only. A recent application of this methodology pertains the restoration of the original morphology of the badly deformed fossil human calvarium of Ceprano [27].

Here, we also propose a solution for dealing with morphological inaccuracy of the retrodeformed model(s) induced by the sole use of landmarks, providing all the routines necessary to retrodeform a triangular mesh based on a bilateral (semi-) landmarks configuration.

We compared our methodology with the RR [16] and NLS [25] methods using a surface mesh representing a cranium of *Gorilla gorilla* that we virtually deformed under controlled conditions. We used this model to compare the performance of the symmetrization procedure proposed here (landmarks + semi-landmarks) to the RR [16] and NLS [25] methods. We further analyzed the effect that the number of bilateral semi-landmarks and the amount of asymmetric noise have on the accuracy of the retrodeformation.

We then applied the retrodeformation procedure to real-world data using the cranium of the Neanderthal specimen Saccopastore 1 (SCP1). SCP1 was discovered in 1929 in the aggradational succession of the Aniene River Valley north of Rome, in Italy; it is chronologically referred to the last interglacial period MIS5e [28], although a MIS7 dating is conceivable [29], even in view of its plesiomorphic features [28]. SCP1 represents a good case-study for our purposes, because of its almost completeness and integrity, combined with well detectable asymmetries in the facial complex and other slight distortions in the cranial base and in the braincase.

## Methods and implementation

Retrodeformation based on a set of bilateral landmarks aims to remove taphonomic deformations that can be interpreted as a series of locally affine deformations. This means that this method is only applicable to cases where the deformations affected the fossil bilaterally—for cases with unilateral defects/compressions this would result in an averaging of this unilateral defect on both sides. It is presumed that these series of deformations can be encoded within the asymmetry of bilateral landmarks, formulating the retrodeformation as a symmetrization. For calculating the retrodeformation, we implemented a closed form solution proposed by Ghosh and colleagues [25] that consists of two steps:

1. Symmetrization of weighted local neighbourhoods [29]
2. Solving for global symmetry while minimizing the deformation in each local neighbourhood

Based on the symmetrized landmark coordinates, the original mesh then is retrodeformed by a TPS deformation [23] calculated from the original and the retrodeformed coordinates. To make this a reasonable deformation, the selected bilateral coordinates should be covering the complete structure: with TPS being a Radial Basis Function (RBF), regions far from landmarks are mostly affected by non-local aspects of the TPS, which might lead to unwanted distortions. Unfortunately, anatomical landmarks are most often not uniformly distributed, so using semi-landmarks on curves and surfaces [30] is required to represent the entire geometry of the structure adequately.

While asymmetry can be assessed reliably for manually placed anatomical landmarks [31], semi-landmarks constituting curves and surface patches might introduce additional asymmetric noise that may lead to asymmetry effects, as there is no objective criterion for their placement. While Ghosh and colleagues [25] and Tallman and colleagues [13] simply suggest using unregularized semi-landmarks placed in Landmark Editor, we tackle the additional problem of asymmetric noise by using a procedure outlined in Schlager [32]. Here a symmetric configuration is calculated by averaging the original and the mirrored landmark configuration.

In order to remove asymmetric noise, the semi-landmarks are allowed to slide along the surface, minimizing bending energy [33] towards this perfectly symmetric configuration. This makes the landmark configuration as symmetric as the actual surface allows it to be and ensures that the retrodeformation only takes care of meaningful asymmetry, discarding noise.

As the algorithm proposed by Ghosh and colleagues [25] rotates and translates the landmarks to their centroid and into the axis of global symmetry, we finally reverse these transformations to allow a direct comparison between the retrodeformed surface/landmarks and the original ones.

Our proposed workflow in R is as follows:

1. Load surface mesh and landmarks
2. Specify correspondences between bilateral pairings between landmarks
3. Specify curve and surface semi-landmarks
4. Relax bilateral semi-landmark configuration against a symmetrical consensus
5. Compute retrodeformation on landmarks (and rotate them back into the original coordinate system)
6. Apply deformation to surface mesh by TPS.

The procedure is available in the R environment embedded in the Morpho R package [26]. The functions can be run in MacOS, Windows and Linux operating system platforms.

### The main functions

We briefly introduce the main functions that are used in the following example.

symmetrize creates a symmetric version of a landmark configuration by mirroring it, rotating it back onto the original using Procrustes registration [34] and averaging both the original and the mirrored version [35].

relaxLM performs the relaxation of one set of landmarks, located on a triangular surface mesh, against another set by minimizing the bending energy between both sets of landmarks [33].

retroDeform3d is the workhorse function and implements the retrodeformation algorithm outlined by Ghosh and colleagues [25]. While we stuck to the default parameters suggested in the original paper, the user is free to adapt those to his own need.

retroDeformMesh is a wrapper combining retroDeform3d and tps3d. It first calculates the retrodeformation based on the bilateral landmarks and subsequently applies a TPS deformation, based on this landmark information, to a given triangular surface mesh.

### Artificial case-study: A deformed cranium of *Gorilla gorilla*

In addition to the modification and implementation of an existing algorithm, making the underlying code freely available to users, we intended to assess a variety of factors contributing to the accuracy of the retrodeformation.

We addressed the following questions:

1. How does the addition of surface semi-landmarks (from 10 to 250 on each side) affect the accuracy of the shape retrieval?
2. How does the method including 100 semi-landmarks (on each side) compare to only using anatomical landmarks?
3. How does asymmetric random noise affect the accuracy?

We compared our procedure described above to the method of RR [16] and NLS [13] using anatomical landmarks only.

To achieve this, we deformed a digital copy of a cranium of *Gorilla gorilla* (USNM 174712 from Smithsonian Institution’s Division of Mammals) but leaving the mesh topology intact, so we can track the accuracy in shape retrieval of each vertex. The controlled deformation was achieved by applying two transformations: at first, the polygons on the left side were expanded, and the polygons on the right side were compressed simulating a “bending” pressure. We then added the simulation of a “twisting” strain by rotating the anterior and posterior portions of the cranium respectively in clockwise and counterclockwise directions.

**Fig 1:**
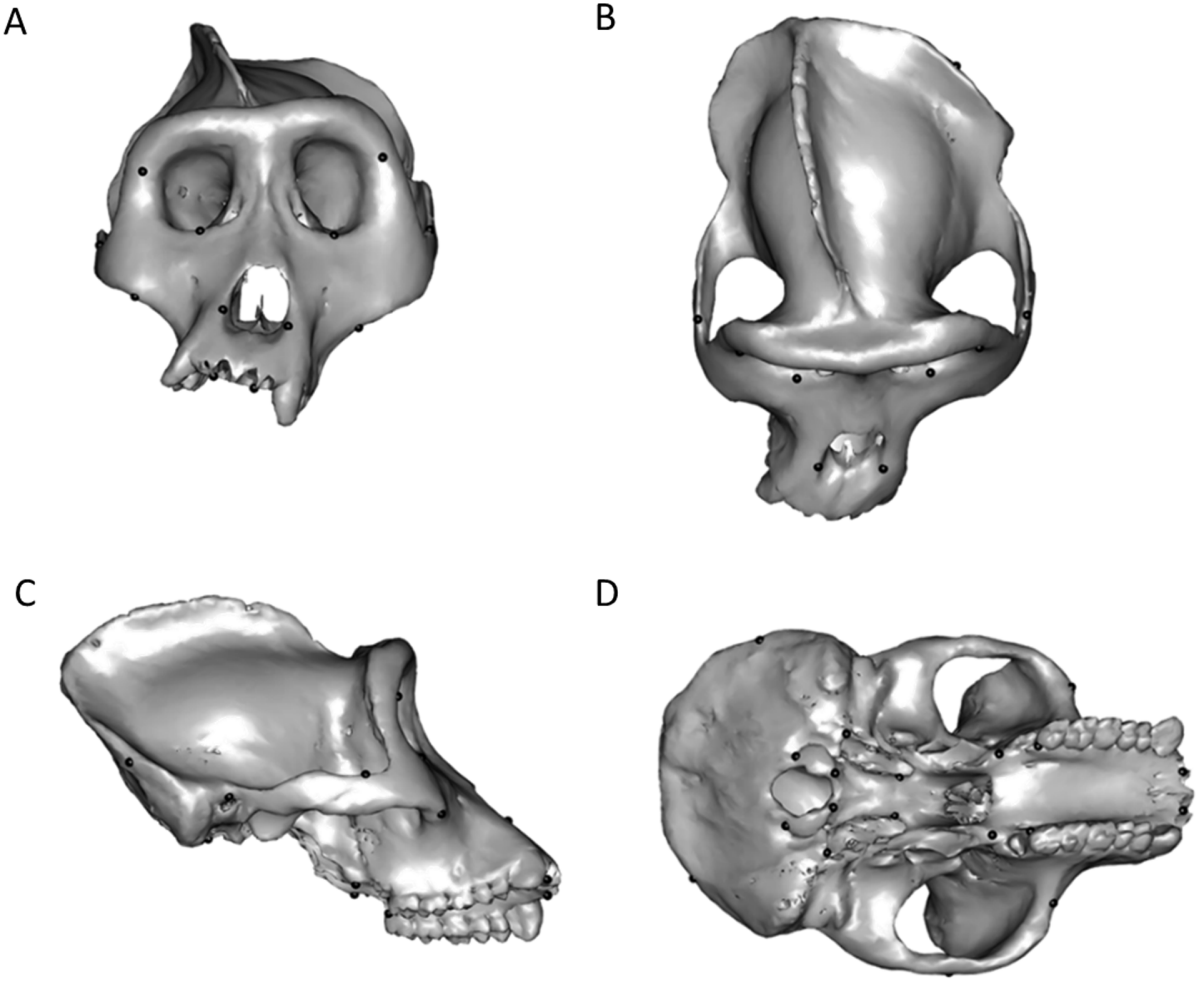
Deformed surface mesh of *Gorilla gorilla* with landmarks. The virtually deformed cranium used as reference for the comparisons of retrodermation methods in frontal (A), superior (B), right lateral (C) and ventral view (D).

To compare the original and the retrodeformed meshes we used Procrustes alignment after placing on the *Gorilla* cranium 14 pairs of landmarks (Fig 1).

We employ average per-vertex displacement as well as average vertex to surface distance, from the retrodeformed to the original mesh, as metrics to assess the error of the shape retrieval.

For assessing the effect that the number of semi-landmarks has on the retrodeformation, we started with anatomical landmarks and subsequently increased the number of sampled surface semi-landmarks adding 10 coordinates at a time using a maximum of 250 semi-landmarks on each side. To reduce the error from placing semi-landmarks on the deformed mesh, we applied the following protocol to sample bilateral semi-landmarks on the original mesh and then transport them to the deformed mesh using barycentric coordinates:

1. Place anatomical landmarks manually
2. Sample equidistant coordinates from the right side only using k-means clustering
3. Rotate them to the right side using the anatomical landmarks
4. Project them onto the original mesh
5. Symmetrize them using the procedure outlined in this article
6. Transfer them to the deformed mesh using barycentric coordinates and the fact that mesh topologies are identical

**Fig 2:**
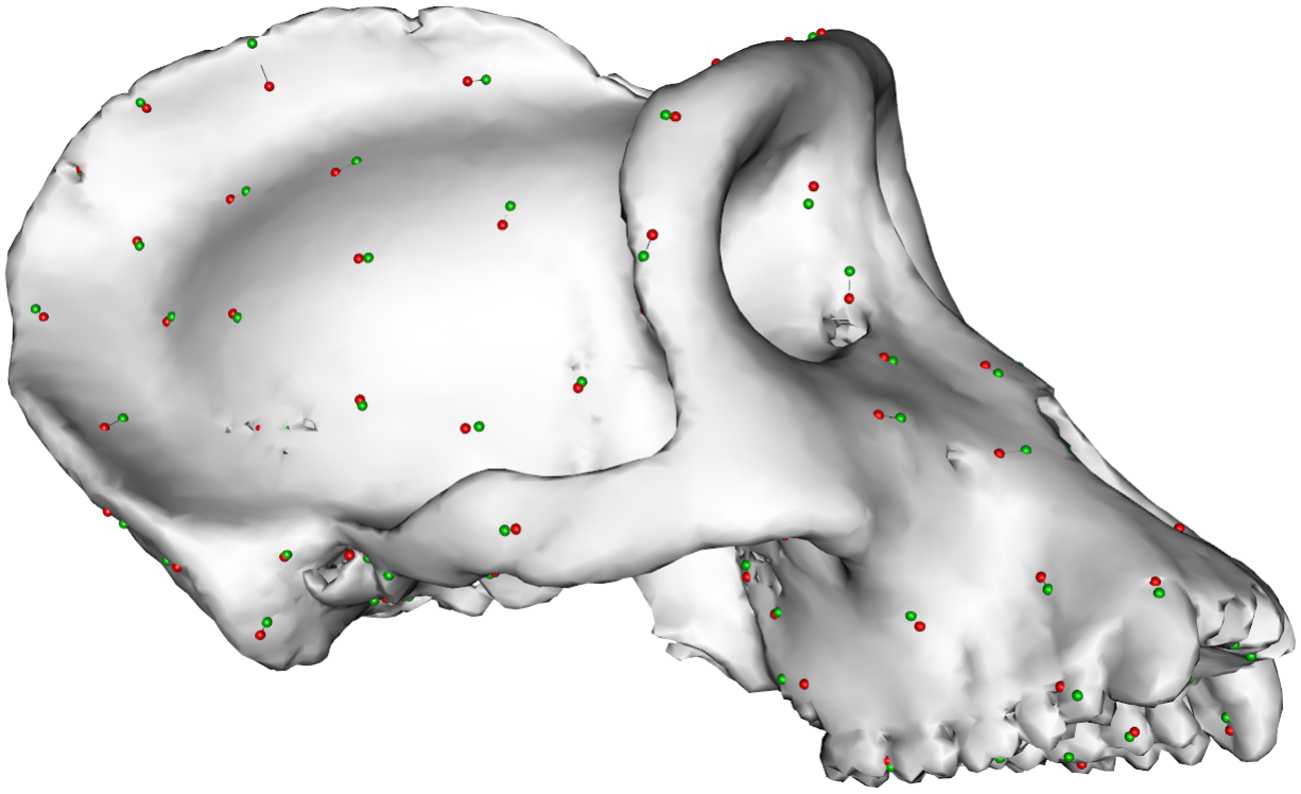
Semi-landmark symmetrization. Visualization of asymmetric noise with a standard deviation of 2 mm. Red: original placement; green: displaced coordinates.

Our proposed method involves a symmetrization step in case bilateral semi-landmarks are used to capture the entire geometry of the structure in question. This additional symmetrization step is intended to deal with asymmetric noise that affects the estimation of the local planes of symmetry. In real world applications it might be hard to determine the correct position of the bilateral counterparts when no anatomical criterion is available, as is generally the case with semi-landmarks. We employed a simulation approach in order to evaluate the effect asymmetric noise has on the retrodeformation’s outcome. First, 100 semi-landmarks were sampled on each side. Asymmetric noise was then simulated by starting with the correct positions and subsequently adding increasing amounts of random noise to the semi-landmark positions along the surface, with the standard deviation of the added noise ranging from 0 to 7.4 mm (cf. Fig 2). We then compared the results with and without our proposed regularization step.

### Real-world case-study: The Neanderthal cranium of Saccopastore 1

To provide a real use case and to demonstrate the software application, we chose the Neanderthal cranium discovered in the site of Saccopastore, in the city of Rome, referred to as Saccopastore 1. SCP1 represents one of the most complete Neanderthal crania available, including the whole base, the vault and large parts of the face, despite some damage that occurred at the time of its accidental discovery in 1929 [28,36,37]. Despite its completeness, it lacks some small portions. The most important damage is represented by the loss of the browridges, broken along their entire extension, exposing the internal surface of the frontal sinuses. This happened at the time of its accidental discovery, when two holes in the vault were also produced. It also lacks the zygomatic arches, while the right malar bone is severely damaged and many dental crowns are missing.

**Fig 3:**
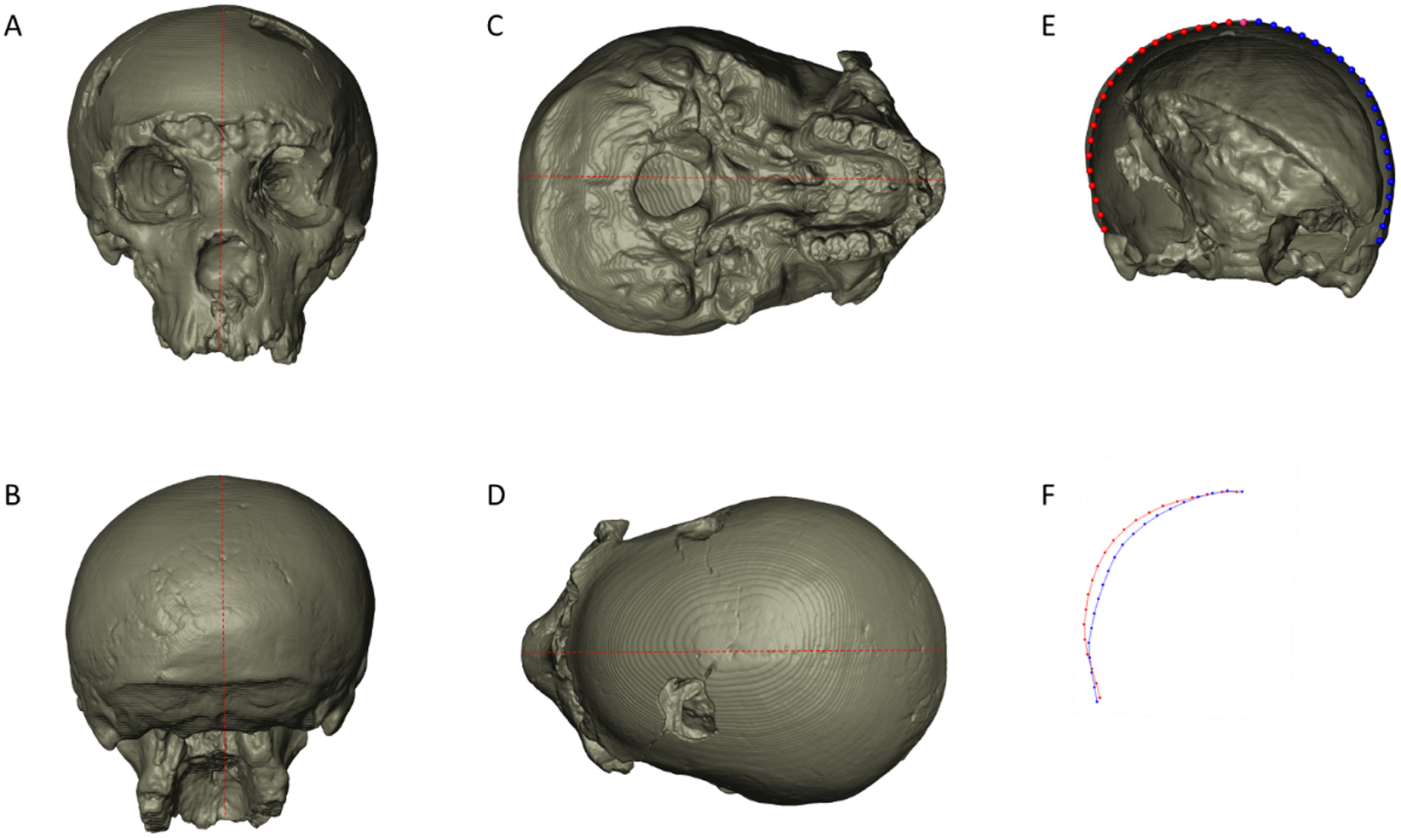
The pattern of deformation in Saccopastore 1 (SCP1). Frontal (A), basal (C), posterior (B) and superior (D) views of the fossil cranium; the red dashed line represents the mid-sagittal plane as defined by the points: supra-glabellar, bregma, lambda. The external outline of a coronal plane corresponding to the biporionic width (E), subdivided in 41 evenly-spaced points. The left set of the external outline is mirrored to emphasize the difference between the two parietal profiles (F).

As it often happens, this fossil cranium shows asymmetries of various entities. Particularly, there is a lateral bulging of the right parietal wall with respect to the left side. In posterior and inferior views, the distortion apparently involves the morphology of the entire nuchal plane, which seems to be rotated toward the left side (as shown by the orientation of both the external occipital crest and the foramen magnum with respect to the mid-sagittal plane), while the left mastoid is by contrast more exposed downward than the right one and the left acoustic meatus is more anterior than the left one (Fig 3).

At the same time, the facial complex of SCP1 appears somewhat rotated with respect to the neurocranium, contributing to the appearance of a peculiar pattern of facial asymmetry, where the right side is more extended towards the mid-sagittal plane (as visible in superior view) and the left side is more exposed downwards, producing a clear inclination of the occlusal plane. The shape and position of the nasal aperture are also clearly asymmetric, consistently with this general pattern of the facial complex.

A surface mesh was generated from CT-scanning of SCP1, of which a downsampled version is included in the R-package as supplementary data. IDAV Landmark editor (http://graphics.idav.ucdavis.edu/research/EvoMorph) was used to record anatomical landmarks as well as supplementary curves and surface patches of semi-landmarks. In particular, we used 86 bilateral landmarks (43 on each side), 6 curves (140 semi-landmarks) and 8 patches (398 semi-landmarks) for 624 anatomical/geometrical points (312 on each side). We included a low-resolution and scaled version of SCP1 in PLY format and the landmarks as ASCII file in .*pts* format specified by the Landmark Editor.

Here we provide a working example using real-world data to regularize the semi-landmarks and subsequently perform a retrodeformation, following the procedure described above.

First, we load the package and read the provided data.

require(Morpho)

require(rgl)

landmarks <- read.pts(system.file("extdata","landmarks.pts",package = "Morpho"))

SCP1 <- file2mesh(system.file("extdata","SCP1.ply",package = "Morpho"))

Now we have to group the landmarks according to their side (left and right) and type (fixed landmarks, semi-landmarks on curves and semi-landmarks on surfaces). The first 86 landmarks are manually placed with the first 43 being on the left hand side and the rest on the right hand side.

fix <- 1:86

nfix <- 86

fixRight <- 1:(nfix/2)

fixLeft <- fixRight+length(fixRight)

Extract the indices of landmarks belonging to individual curves and patches from the rownames of the matrix containing the landmarks.

leftRighInfo <- cExtract(landmarks)

In our example, odd numbered curves and surface patches are located on the right hand side

curveLeft <- c(leftRighInfo$C000,leftRighInfo$C002,leftRighInfo$C004)

curveRight <- c(leftRighInfo$C001,leftRighInfo$C003,leftRighInfo$C005)

curves <- leftRighInfo[grep("C",names(leftRighInfo))]

patches <- unlist(leftRighInfo[grep("P",names(leftRighInfo))])

patchesLeft <- c(leftRighInfo$P000, leftRighInfo$P002, leftRighInfo$P004, leftRighInfo$P006)

patchesRight <- c(leftRighInfo$P001, leftRighInfo$P003, leftRighInfo$P005, leftRighInfo$P007)

Now we create a matrix with left column containing the row indices of the landmarks placed on the left hand side and the right column contains their corresponding right-hand landmarks

pairedLM <- cbind(c(fixLeft, curveLeft, patchesLeft), c(fixRight, curveRight, patchesRight))

Based on this information we create a perfectly symmetric landmark configuration.

landmarksSym <- symmetrize(landmarks,pairedLM)

**Fig 4:**
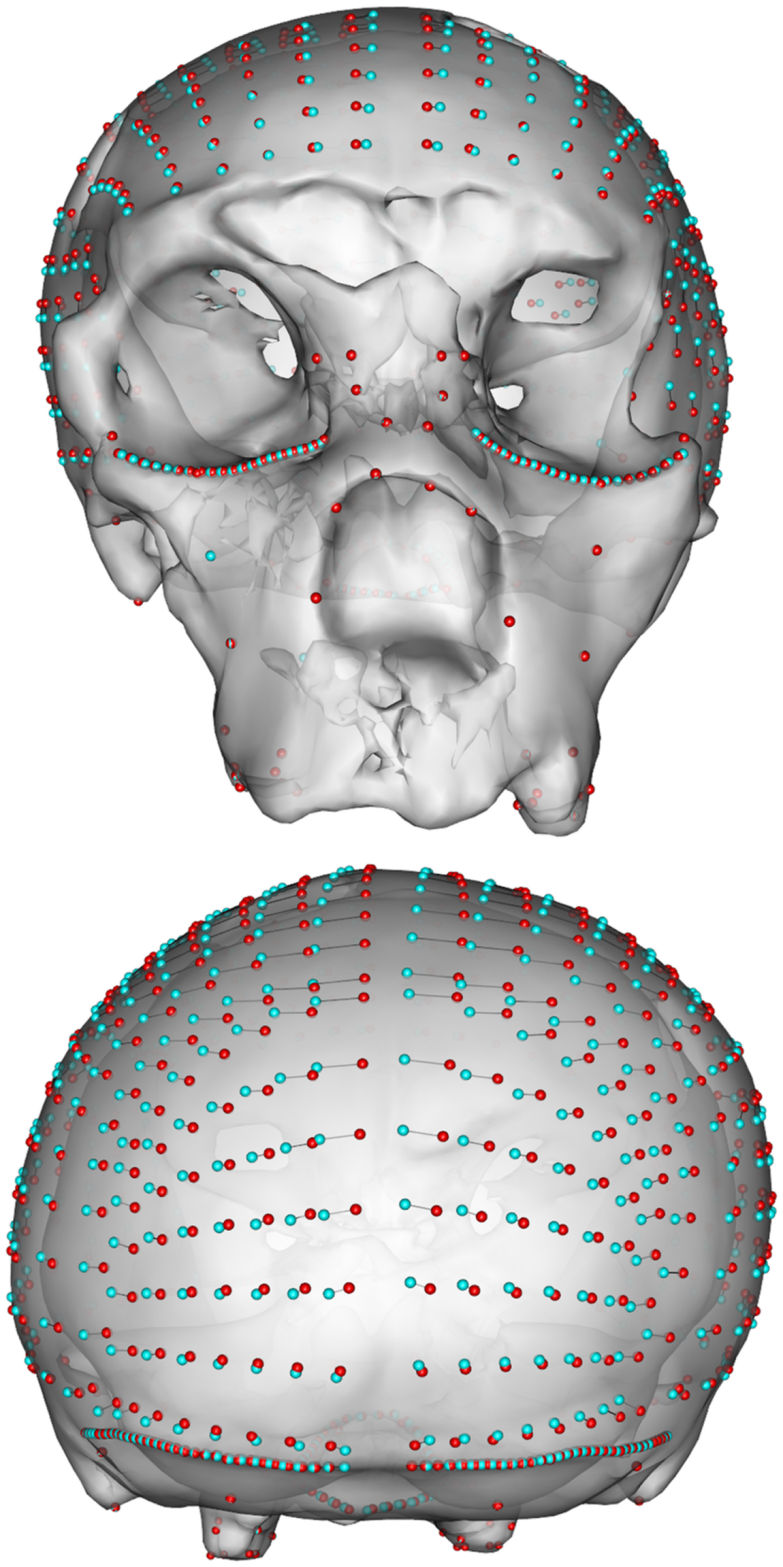
Original surface mesh of SCP1 with landmarks. The landmark sets before (red) and after (cyan) the retrodeformation procedure shown in frontal and posterior views.

We relax the semi-landmarks against this symmetric version to remove all asymmetry not present in the actual data, thus making the landmark configuration as symmetric as the surface geometry allows and visualize the effect (Fig 4).

landmarkSlide <- relaxLM(landmarks,landmarksSym,SMvector = fix,outlines = curves,mesh = SCP1,iterations = 3,surp = patches,deselect = T)

deformGrid3d(landmarkSlide, landmarks,col2 = 5)

shade3d(SCP1,col = "white",alpha = 0.7)

Finally, we use the function retroDeformMesh, that performs steps 5 and 6 and also rotates the data back into the original coordinate system. The function is basically a wrapper for the underlying functions retroDeform3d and tps3d. The function accepts a surface mesh of class mesh3d, a *k × 3* matrix of bilateral landmark coordinates and an *l × 2* matrix identifying the bilateral counterparts of each landmark.

SCPdeform <- retroDeformMesh(SCP1,landmarkSlide,pairedLM = pairedLM)

This command returns a named list with $mesh being the deformed mesh and $landmarks a list containing the original ($orig) and the retrodeformed ($retrodeformed) landmarks.

**Fig 5:**
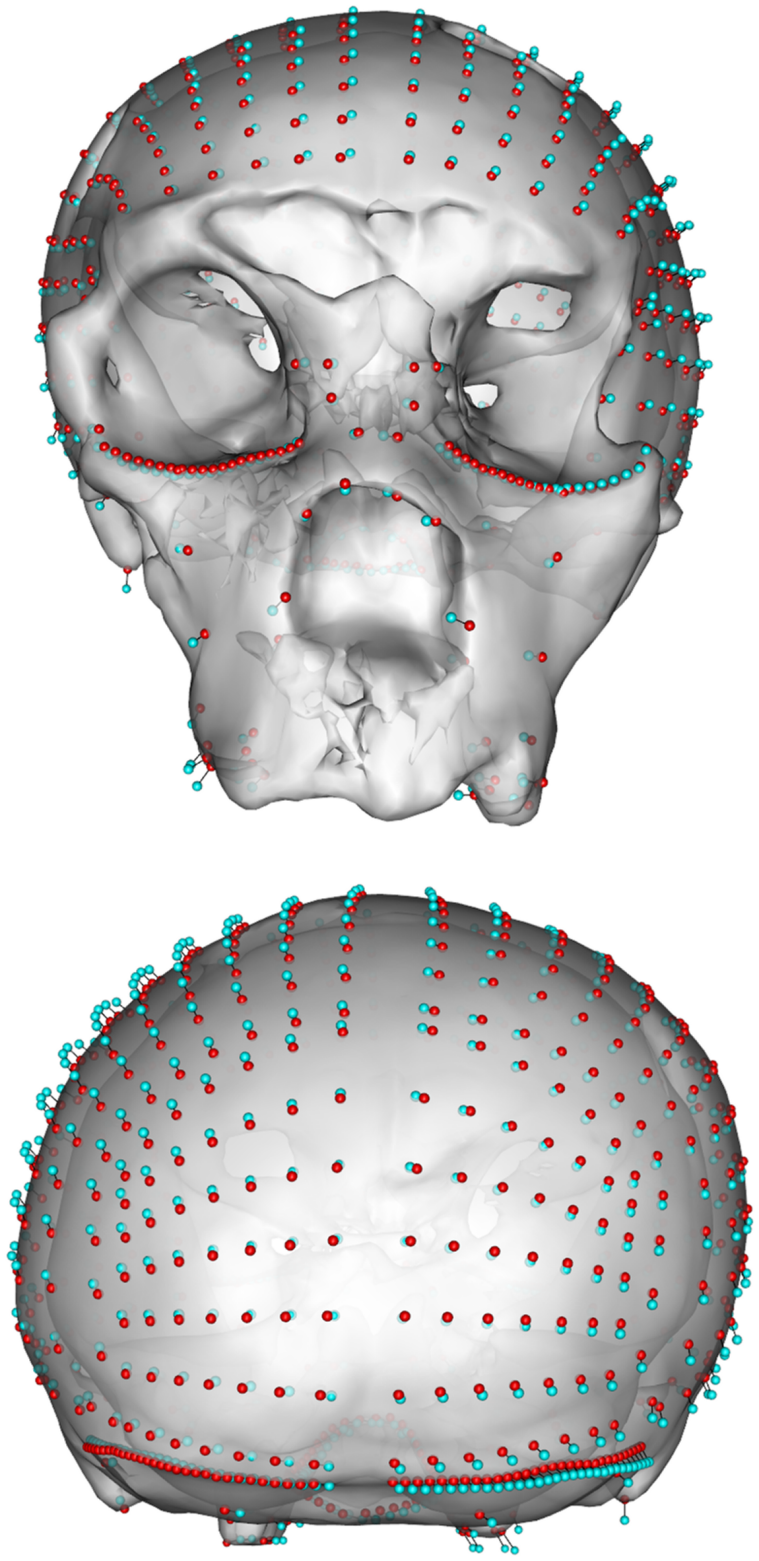
Removal of asymmetric noise in the semi-landmarks. Placement of semi-landmark sets before (cyan) and after sliding (red) on the cranium of SCP1 in frontal and posterior views.

Now we visualize the landmark displacement (Fig 5) calculated by the retrodeformation and add the original surface for better comparison.

deformGrid3d(SCPdeform$landmarks$orig,SCPdeform$landmarks$deformed,col2 = 5)

shade3d(SCP1,col = "white",alpha = 0.7)

**Fig 6:**
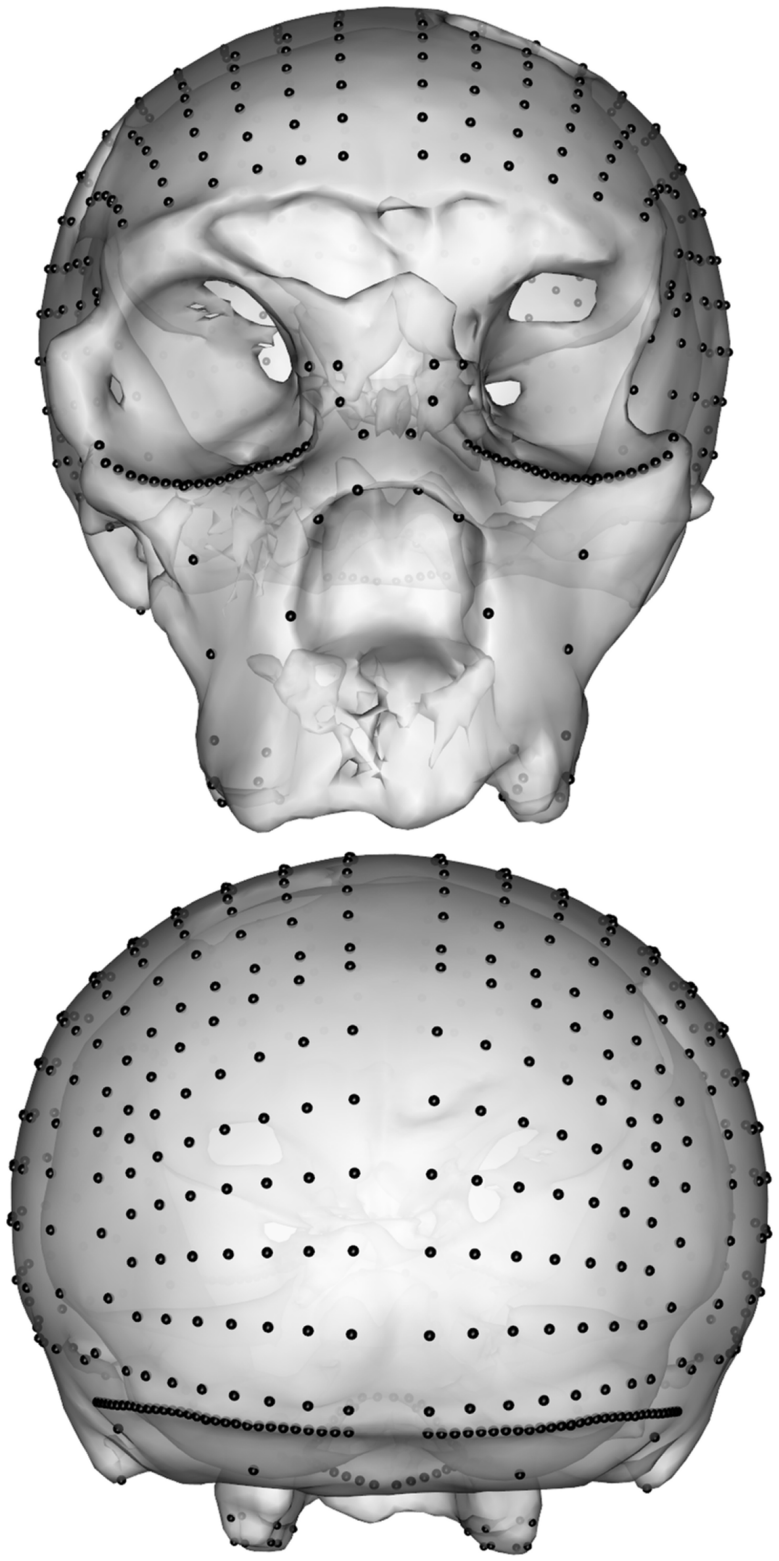
The surface mesh of SCP1 warped to the new (semi-) landmark positions.

Finally, we visualize the deformed surface plus the retrodeformed landmarks (Fig 6).

wire3d(SCPdeform$mesh,col = 3)

spheres3d(SCPdeform$landmarks$deformed)

**Fig 7:**
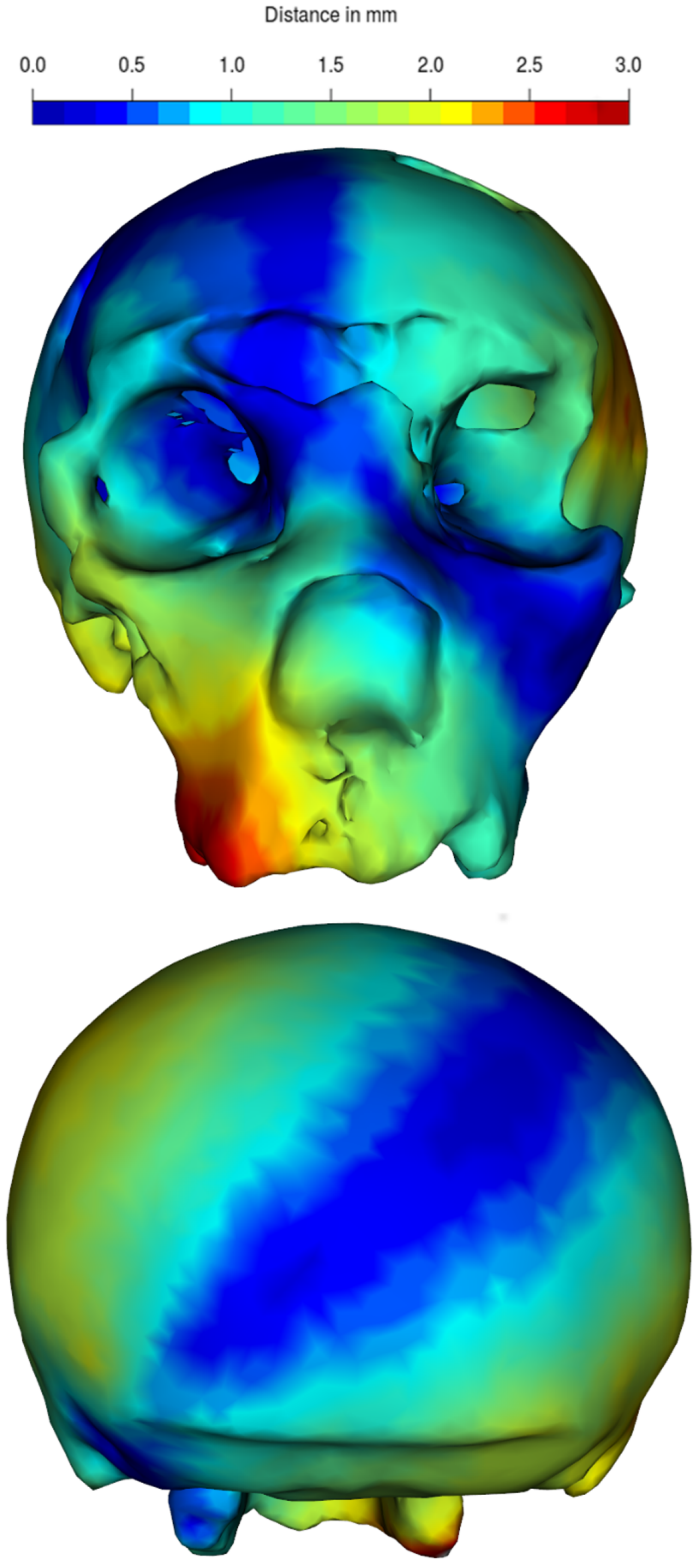
Visualization of the per-vertex displacement between original and retrodeformed mesh of SCP1. The mesh distance expressed in millimeters (mm) ranging from 0 (blue) to 3.0 (red).

Additionally, we visualize the vertex displacement of the surfaces by creating a distance map (Fig 7).

dists <- apply(vert2points(SCPdeform$mesh)-vert2points(SCP1),1,base::norm,"2")

meshDist SCPdeform$mesh, distvec = dists, from = 0,to = 3)

## Results

### Retrodeformation of the artificial deformation case-study using a Gorilla cranium

**Fig 8:**
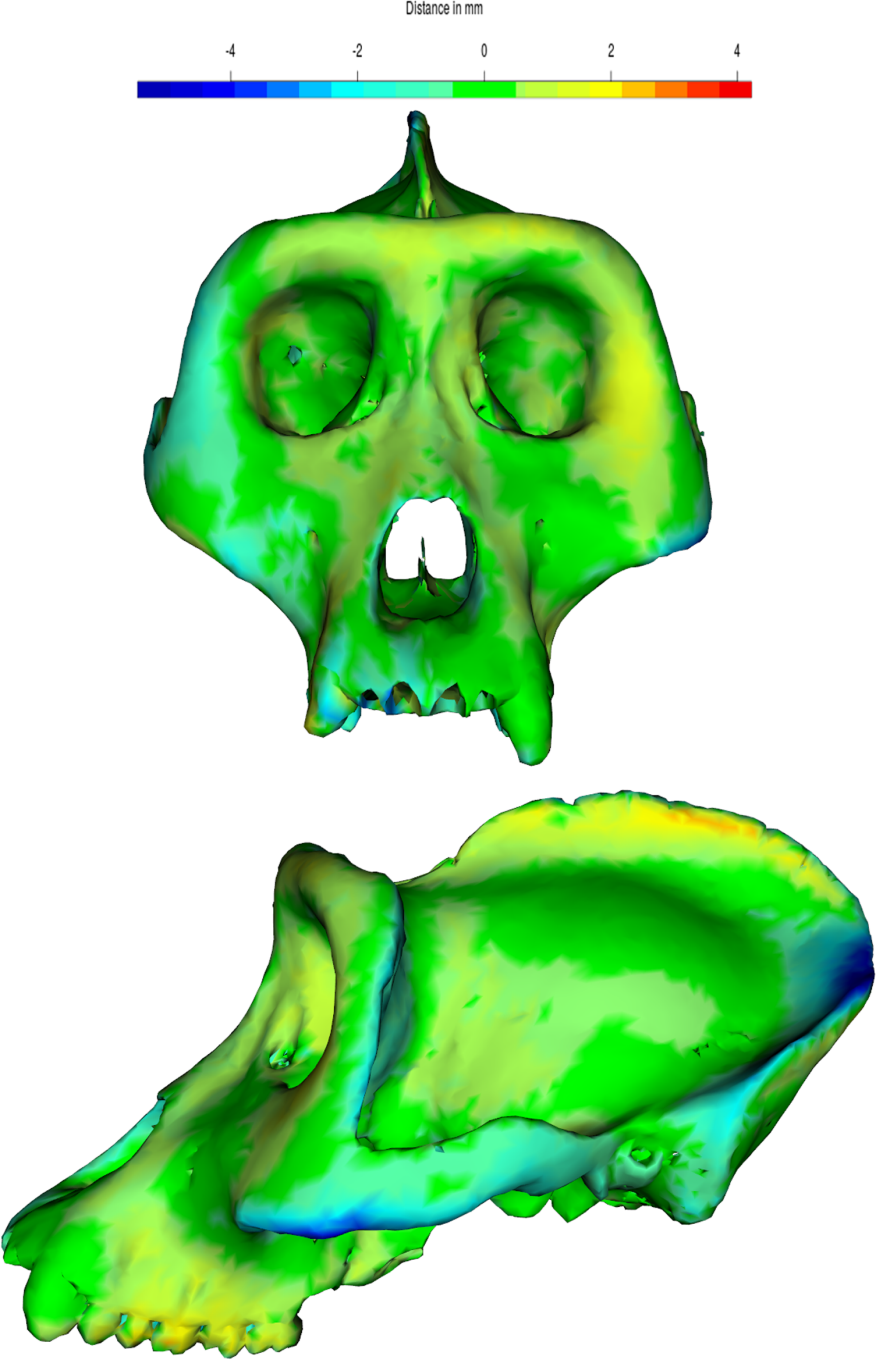
Visualization of the retrodeformation error using our proposed method in the Gorilla case study. The heat-map shows the surface to surface distance between the retrodeformed and the original Gorilla cranium.

The average distance between the surfaces of the original model and the deformed model amounts to 3.53 mm (maximum 27.94 mm) with an average per-vertex displacement of 7.78 mm (maximum 34.28 mm). The application of the three methods applied in this study decreased the average distances respectively to 1.42/3.62 mm (RR), 2.0/3.47 mm (NLS using landmarks only) and 0.73/1.8 mm (our proposed procedure using 100 semi-landmarks on each side; result can be seen in Fig 8). Regarding the maximum distances, this resulted in 18.95/25.18 mm (RR), 21.03/20.05 mm (NLS using landmarks only) and 7.13/10.12 mm for our suggested procedure.

**Fig 9:**
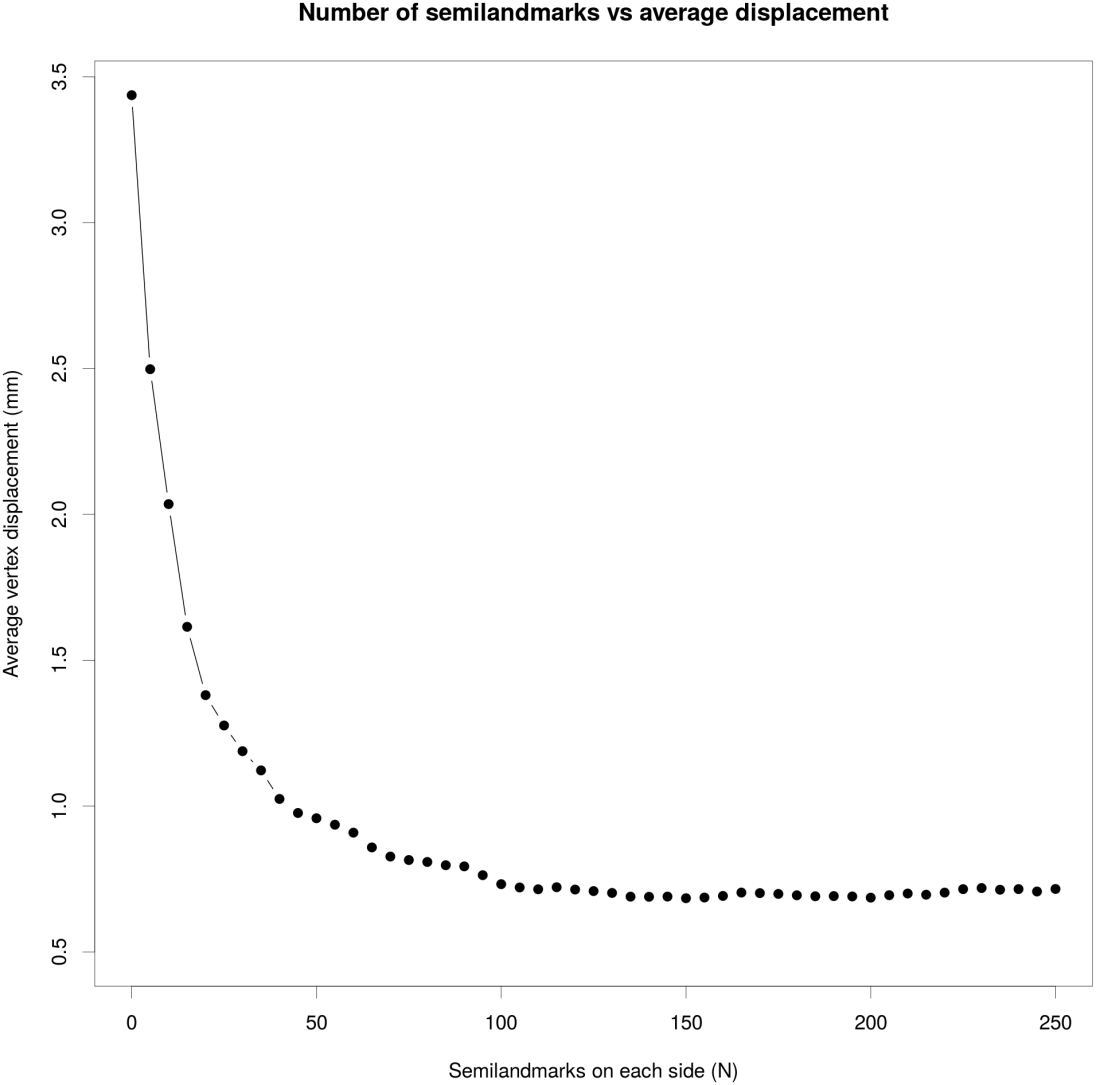
Relation between number of semi-landmarks and recovery of anatomical information (Gorilla case study). In the plot are reported the number of semilandmark per side (x axis) used at each iteration and the respective amount of vertex displacement (y axis) after the retrodeformation procedure.

Testing the effect of adding semi-landmarks, we noticed a strong improvement of adding bilateral surface coordinates (Fig 9). Adding 50 coordinates on each side already reduces the average vertex displacement from 3.5 mm to 1.05 mm and adding another 100 coordinates decreases it further to 0.68 mm. Adding more than 100 coordinates per side does not seem to further minimize the error significantly.

**Fig 10:**
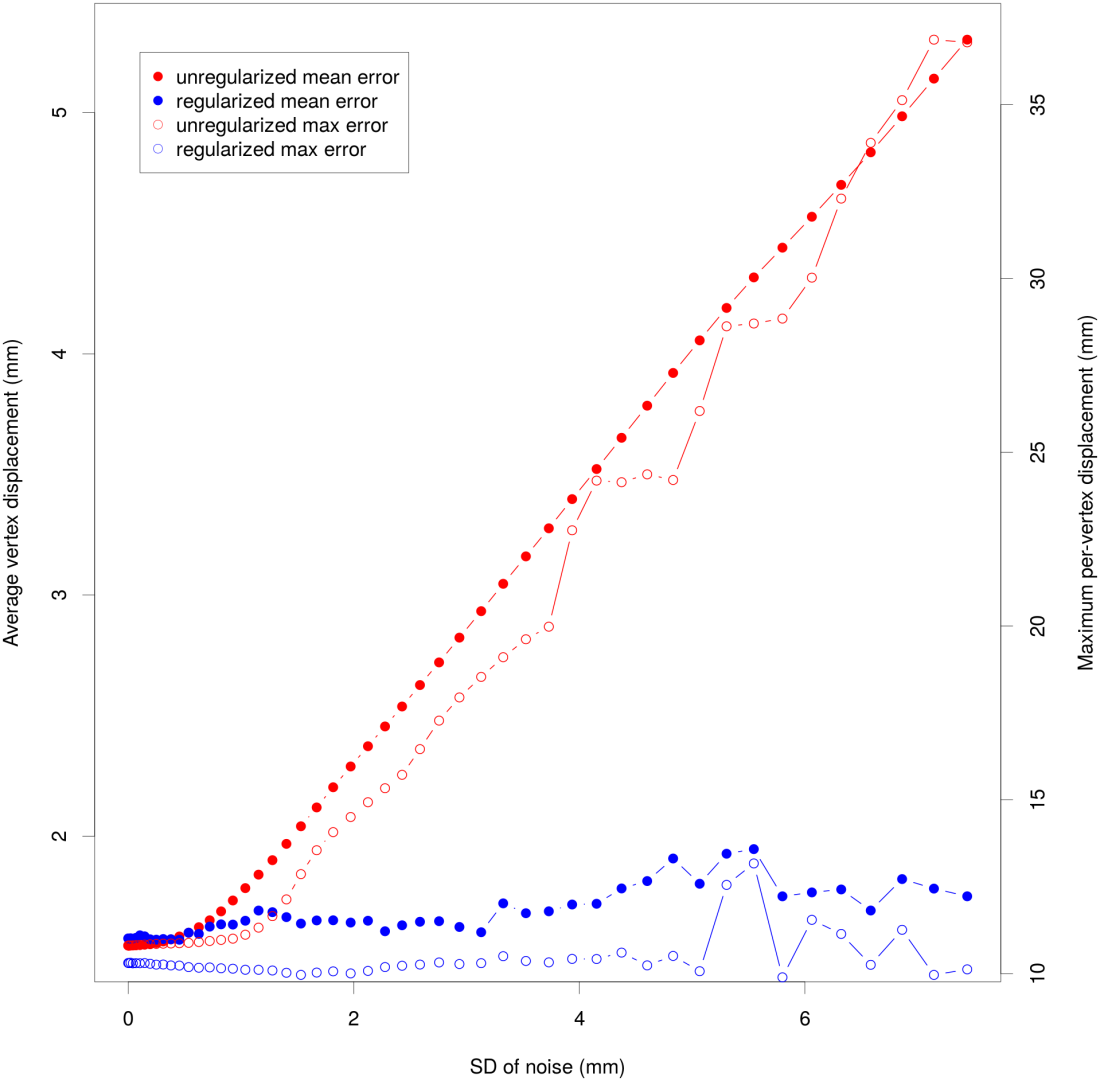
Effect of asymmetric noise on retrodeformation error (Gorilla case study). Using 200 semi-landmarks, this figure shows the effect of noise in regard to mean and maximum per-vertex error. Blue solid dots: mean error of our proposed regularized method; Mean (solid circles and left y axis) and maximum (open circles and right y axis) error of proposed regularized method (blue) and unregularized retrodeformation (red).

Our suggested additional step for processing semi-landmarks is intended to remove asymmetrical noise that may lead to false estimations of the local planes of asymmetry and thus compromising the resulting deformation. While small amounts of noise have no adverse effects to the result, the necessity of the regularization step becomes evident for noise with a standard deviation > 2 mm (Fig 10). The unregularized approach leads to a constant increase of error but the regularization step keeps the average error oscillating below 2 mm and the maximum error below 15 mm, even with large amounts of noise present.

### Retrodeformation of Saccopastore 1

The result of each step of the proposed symmetrization method applied to SCP1 is shown in Figs 4–7. The changes observed after the symmetrization procedure are expressed also through the calculation of the linear distance between a selection of bilateral landmarks before and after the application of the protocol. The application of the proposed methodology to this specimen resulted in a retrodeformation of the cranium associated with complete symmetrization (Figs 5–7). The procedure completely removed the asymmetry affecting the neurocranium producing a full realignment of the parieto-temporal region and carrying a counterclockwise rotation of the facial complex with respect to the neurocranium. The main differences between the original cranium and the retrodeformed ones are visible in Fig 7 and the Euclidean distances between paired sets of landmarks are reported in Table 1.

**Table 1:** Euclidean distances (mm) acquired before and after the retrodeformation procedure between selected paired bilateral landmarks placed on SCP1.

Dist	Original (mm)	Retrodeformed (mm)	Var (%)	Definition
9–52	97.83	98.95	-1.14	The minimum distance between the two temporal lines
41–84	32.00	30.86	+3.56	The maximum breadth of the piriform aperture
29–72	68.30	68.52	-0.33	The distance between the two orbital points
35–78	24.87	23.80	+4.32	The breadth across the nasal space from dacryon to dacryon
10–53	120.25	122.10	-1.54	The bi-porionic breadth
12–55	99.44	100.36	-0.93	The distance between the mastoid tips
20–63	27.92	26.93	+3.54	The maximum breadth of the foramen magnum
13–56	16.47	14.96	+9.16	The basioccipital breadth along the spheno-occipital suture
15–58	49.57	49.02	+1.10	The bi-carotid (temporal bone) breadth
27–70	35.78	34.87	+2.56	The distance between the two pterygoideal hamuli

## Discussion and conclusion

Fossils that are deformed by taphonomic processes are common in the fossil record, and in many cases, deformed specimens are excluded from the sample under investigation [38]. Our proposed procedure is an extension of the method proposed by Ghosh and colleagues [25]. Compared to other existing methods, it allows the investigator to symmetrize a 3D model of a biological object not only making use of anatomical points (landmark) but also bilaterally placed semi-landmarks placed on curves and surfaces. That way, the correction for asymmetry due to taphonomic events takes into account local asymmetries throughout the entire 3D model. The resulting deformation is then applied to the complete structure using a TPS interpolation. This procedure allows the retrodeformation of fragmented fossil specimens by compensating for the sparsity of anatomical landmarks by using bilaterally placed semi-landmarks along curves and on surfaces [27]. The application of a non-linear symmetrization approach becomes crucial in all those cases in which the taphonomic deformation cannot be viewed as one globally affine compression.

As tested in the case study represented by the *Gorilla* cranium digitally deformed under controlled conditions, our proposed procedure is a straight-forward combination of sliding semi-landmarks with the GM toolkit for assessing asymmetry in landmark data. Observer error with respect to asymmetry is minimized by allowing the semi-landmarks to slide along the surface to find the position of least bending energy with regard to a perfectly symmetric shape, calculated from the landmarks and their mirrored and relabeled counterparts. Bilateral semi-landmarks without correction for asymmetric noise will most likely result in faulty estimations of the local planes of symmetry and finally to a flawed retrodeformation result. In this paper, we demonstrated that our implementation allows a safe usage of semi-landmarks that significantly improve the retrodeformation accuracy. The added regularization step reduces the noise in the semi-landmarks’ placement, leading to stable results even with extreme levels of asymmetric noise added.

As semi-landmarks have no biological measure of homology, manual or semi-automatic bilateral placement of these coordinates might result in unwanted asymmetry effects not present in the actual structure [32]. As the asymmetry captured by landmarks can be attributed to both actual asymmetry and observer error, minimization of the latter is essential for obtaining reliable results.

The application of the procedure described in this work to the real case study of the Neanderthal cranium (SCP1) has led both the reduction of the asymmetry of the neurocranium, the counterclockwise rotation of the facial complex (detectable in frontal view, Fig 7) and the shrinkage and expansion of the neurocranium detected by the bi-temporal lines diameter and the bi-carotid breadth respectively (Table 1). Also, we detected small-scale changes in the retrodeformed version of SCP1 as the size of the breadth of the nasal and basioccipital regions (Table 1).

To conclude, in this paper we proposed and presented step by step functioning and use of a non-linear symmetrization method of retrodeformation with the aid of bilateral landmarks and semi-landmark sets. The advantage of the application of this method is the detection of the locally affine deformation scattered all over the cranium surface reducing the interpolated symmetrization performed by TPS.

We showed the benefit of adding semi-landmarks to capture the geometry of surfaces without proper anatomical landmarks. Our proposed regularization step proved to be relevant in cases with asymmetric noise. The R code to perform the retrodeformation procedure, the 3D model and the (semi-) landmark sets are available and stored in the R-package Morpho [26], as an extension of the R Statistical computing environment [39] and the protocol is easily replicable.

## Supporting information

S1 Comparative Code This supplementary material provides the code and data to reproduce the results from our artificial case study using a virtually deformed *Gorilla gorilla* cranium. (ZIP)
